# Electrospun silk-based nanofibrous scaffolds: fiber diameter and oxygen transfer

**DOI:** 10.1007/s40204-016-0046-6

**Published:** 2016-02-09

**Authors:** Masoud Dadras Chomachayi, Atefeh Solouk, Hamid Mirzadeh

**Affiliations:** 1 Polymer Engineering and Color Technology Faculty, Amirkabir University of Technology (Tehran Polytechnic), Tehran, Iran; 2 Biomedical Engineering Faculty, Amirkabir University of Technology (Tehran Polytechnic), Tehran, Iran

**Keywords:** Wound dressing, Silk fibroin, Electrospinning, GMDH, ANN, Oxygen profile

## Abstract

In this study, silk fibroin was extracted from cocoons of silkworms and fabricated into nonwoven mats by electrospinning method. A new model based on the group method of data handling (GMDH) and artificial neural network (ANN) was developed for estimation of the average diameter of electrospun silk fibroin nanofibers. In this regard, concentration, flow rate, voltage, distance, and speed of collector were used as input parameters and average diameter of the fibers was considered as output parameter. Two models were capable to estimate average diameter of fibers with good accuracy. The average absolute relative deviation for GMDH and ANN models was equal to 3.56 and 2.28 %, respectively. Furthermore, due to importance of oxygen delivery to site of injury to promote wound healing, continuity equation for mass transport was employed for prediction of oxygen profile in the system containing wound dressing and skin. The result showed that our prepared wound dressing is capable to pass the oxygen completely to the skin layer and is not acting as a barrier for oxygen delivery to wound site. Since average nanofibers diameter can influence the mat physical, mechanical and biological properties then this model may serve as a useful guide to obtain tailor made and uniform silk nanofibers at various combinations of process variables.

## Introduction

Nanotechnology is a growing field of manufactured materials with sizes less than 1 μm, and it is particularly useful in the field of biology because these applications replicate components of a cell’s in vivo environment. Nanofibers, which mimic collagen fibrils in the extracellular matrix (ECM), can be prepared from a host of natural and synthetic biomaterials and have multiple properties that may be beneficial to field of materials for medical applications. These properties include a large surface-area-to-volume ratio, high porosity, improved cell adherence, proliferation and migration, and controlled in vivo degradation rates. The large surface area of nanofiber mats allows for increased interaction with compounds and provides a mechanism for sustained release of antibiotics, analgesics, or growth factors into injured tissue; high porosity allows diffusion of nutrients and waste. Improved cell function on these scaffolds will promote healing. Controlled degradation rates of these scaffolds will promote scaffold absorption after its function is no longer required (Hromadka et al. 2008). All the mentioned properties can be influenced by fiber morphology and diameter (Min et al. 2004; Kim et al. 2003). It is mentioned in the literature that, those fiber morphology and diameter are depend on many parameters which can be divided into four main categories: polymer properties (molecular weight and solubility), polymer solution parameters (polymer concentration, solution viscosity, conductivity, surface tension, and etc.), processing conditions (applied voltage, nozzle-collector distance, feed rate, and needle diameter), and ambient parameters (temperature, atmosphere pressure, and relative humidity) (Nasouri et al. 2012).

Silk fibroin, derived from silkworm cocoons, has attracted the scientific community due to its good biocompatibility along with suitable mechanical property. Fibroin protein is the major constituent 75 % of the cocoon and the remaining 25 % is sericin protein (Kim et al. 2005; Chirila et al. 2013). The molecular orientation makes this protein form a semi-crystalline structure which contains two phases: highly crystalline antiparallel β-sheet structure and non-crystalline part (Wang et al. 2004). The crystalline part lead to increase the strength and toughness and the non-crystalline part contributes the flexibility and elasticity to the fiber (Jin et al. 2005). Over the past years, many studies have explored silk fibroin in various forms from its regenerated solution, including porous scaffolds, films and electrospun fibers (Wharram et al. 2010). The electrospun silk fibers with uniform micro or nano scale fibers have found utility in producing different biomaterials like wound dressing or scaffolds for a variety of biological applications such as bone, nerve and skin tissue (Valenzuela et al. 2012).

The lack of comprehensive and predictive models is sensing in the field of electrospinning. For instance, it can be characterized that when one of the parameters of electrospinning process is changed what will happen for diameter of nanofibers. The experimental observations show an increase or decrease in fiber diameter. But, it is not possible to predict the diameter magnitude without conducting experimental procedure. Consequently when a special diameter of fibers is required, it is necessary to change various parameters. However, experimental investigations are time consuming and almost expensive. In these situations, existence of predictive models will be so beneficial. Recently, studies have been carried out to determine the feasibility and to optimize the diameter of electrospun nanofibers with different type of mathematical relationships or models such as design of experiments or artificial neural network (Malallah and Nashawi 2005).

Artificial neural networks (ANN) are flexible modeling method which shown excellent performance for modeling different problems. Generally, ANNs are empirical mathematical tools which can model various data sets even in the cases that complex relation is existed between input and output parameters. This method provides flexible non-linear mathematical function mapping of a set of input variables into the output of network. Although ANNs are accurate systems for modeling different problems, but they have a disadvantage about their mathematical structure (Atashrouz et al. 2014a, b). Obtained mathematical structures for an ANN model are complicated and practical application of this type of models is not easy. To address this issue, group method of data handling type neural networks (GMDH-NN) was proposed. GMDH-type neural networks also known as polynomial neural networks has both accuracy in modeling and simplicity in mathematical structure (Atashrouz et al. 2014a, b; Dodangeh et al. 2014).

In this work, GMDH and ANN models are used for prediction of fiber diameter and also comparison of their performance as two different types of neural networks to prepare electrospun nanofibers from silk fibroin. In this regard, concentration, flow rate, voltage, distance, and speed of collector were used as input parameter and diameter of fibers was considered as output parameter. In addition, due to importance of oxygen delivery to site of injury and to promote wound healing process, it will be interesting to predict the oxygen profile within the electrospun mat and also the skin layer as an example. In this regard, a mathematical relationship based on a finite difference method was applied for predicting profile of oxygen in system containing electrospun mat and skin.

## Materials and methods

### Preparation of silk fibroin

Silkworm cocoons were obtained from Gilan University (Rasht, Iran). First, cocoons of *Bombyx mori* were boiled for 45 min in an aqueous solution of 0.02 M sodium carbonate, and then rinsed with distilled water to completely remove sericin. After drying, the degummed silk fibers were dissolved in 9.3 M LiBr at 60 °C for 4 h and then were dialyzed in a dialysis bag (12,000 MWCO, Sigma, USA) against distilled water for 3 days with changing water several times for the removal of the salt. Finally, the film of silk fibroin solution was prepared (Mirahmadi et al. 2013; Uttayarat et al. 2012).

### Electrospinning

Silk-based electrospun scaffolds were fabricated as follows: 10 and 12 % (w/v) solution of silk fibroin in formic acid (98–100 %, Merck) were prepared and stirred at room temperature for 1 h to obtain complete dissolution. The solutions were filled into a 2.5 mL plastic syringe (18–G). Flow rate, distance, speed of collector and spinning voltage were selected in the range of 0.1–0.6 (cc/h), 8–12 (cm), 200–2500 (rpm) and 12–30 (kV), respectively.

### Characterization of the prepared electrospun mat

#### Morphological observation by scanning electron microscopy (SEM)

The morphological investigation of the electrospun nanofibers was performed with scanning electron microscope (AIS2100, South Korea). The intact samples coated with gold for SEM witnessing. In the SEM photos, the fiber diameters were determined by means of Image J software and the results were given as the average diameter ± standard deviation.

#### FT-IR spectroscopy

FT-IR spectroscopy (Tensor 27, Brucker Optics, Germany) was used to determine the protein conformation in electrospun nanofibers of silk fibroin. The scan was collected under absorbance mode from 4000 to 400 cm^−1^ at 4 cm^−1^ scan resolution.

### Group method of data handling (GMDH)

In the GMDH type neural network algorithm, by combination of more than two variables at a time a polynomial expression can be obtained for studied problem. The algorithm is tried to find most appropriate configuration of polynomials which best accuracy. The following series is the form of grand correlation multinomial in GMDH systems (Atashrouz et al. 2015a, b):

1 $$ y_{0} = a_{0} + \sum\limits_{i = 1}^{M} {\sum\limits_{j = 1}^{m} { \ldots \sum\limits_{k = 1}^{M} {a_{ij \ldots k} x_{i}^{n} x_{j}^{n} , \ldots ,x_{k}^{n} \quad n = 0,1, \ldots ,2^{l} } } } $$

where *y*_0_ is the output, *x*_*i*_, *x*_*j*_, and *x*_*k*_ are input parameters and *l* is the number of layers. In addition, *a*_0_, *a*_*ijk*_ are coefficients of grand correlation multinomial which should be adjusted based on the algorithm.

**Fig. 1:**
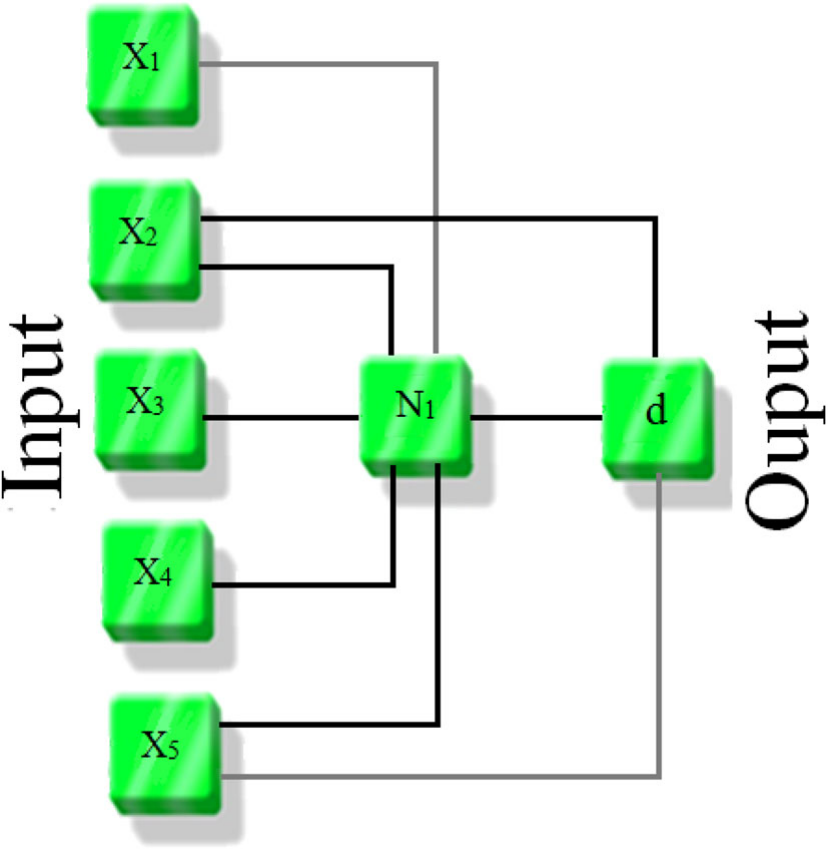
Schematic of proposed GMDH model

Now for an input vector of *x* = (*x*_1_, *x*_2_, …, *x*_*n*_) the output of the multinomial is $f_{i} (x_{1} ,x_{2} , \ldots ,x_{n} )$. The parameters of Eq. 1 should be adjusted based on the real data. It is supposed that real data are show with *r*_*i*_. So, to optimize the grand correlation multinomial, square of difference between real data and calculated data of multinomial expression should be close together:

2 $$ \sum\limits_{i = 1}^{M} {[f_{i} (x_{1} ,x_{2} , \ldots ,x_{n} ) - r_{i} ]}^{2} \to \hbox{min} $$

where *M* is corresponds to the number of observations. Figure 1 shows a schematic of proposed GMDH model. For more detailed information about the group method of data handling, the reader is referred to the literatures (Atashrouz et al. 2015a, b).

### Artificial numeral network (ANN)

Neural networks consist of arrays of simple active units linked by weighted connections. ANN consists of multiple layers of neurons arranged in such a way that each neuron in one layer is connected with each neuron in the next layer. The network used in this study is a multilayer feed forward neural network with a learning scheme of the back-propagation (BP) of errors and the Levenberg–Marquardt algorithm for the adjustment of the connecting weights. Neurons are the fundamental processing element of an ANN, which are arranged in layers that make up the global architecture. The ANN input is the first layer in the network through which the information is supplied. The number of neurons in the input layer depends on the network input parameters. Hidden layers connect the input and output layers. Hidden layers enrich the network for learning the relation between input and output data. In theory, ANN with only one hidden layer and enough neurons in the hidden layer, has the ability to learn any relation between the input and output data (Zhang et al. 1998). Transfer function is the mathematic function that determines the relation between neuron output and the network. The sigmoid transfer function is as follow:

3-a $$ O_{Pj} ({\text{net}}) = \frac{1}{{1 + e^{{ - {\text{net}}}} }} $$

3-b $$ {\text{net}} = \sum\limits_{i = 0}^{n - 1} {w_{i} x_{i} } $$

which “*n*” in Eq. (3) is the number of inputs to the neuron. “*w*_*i*_” is the weight coefficient corresponding to the input “*x*_*i*_” and “*O*_*pj*_” is the output corresponding to the “*j*” neuron. For completion of this section, we illustrate the learning BP algorithm (Atashrouz et al. 2013).

#### ANN training algorithm

**Fig. 2:**
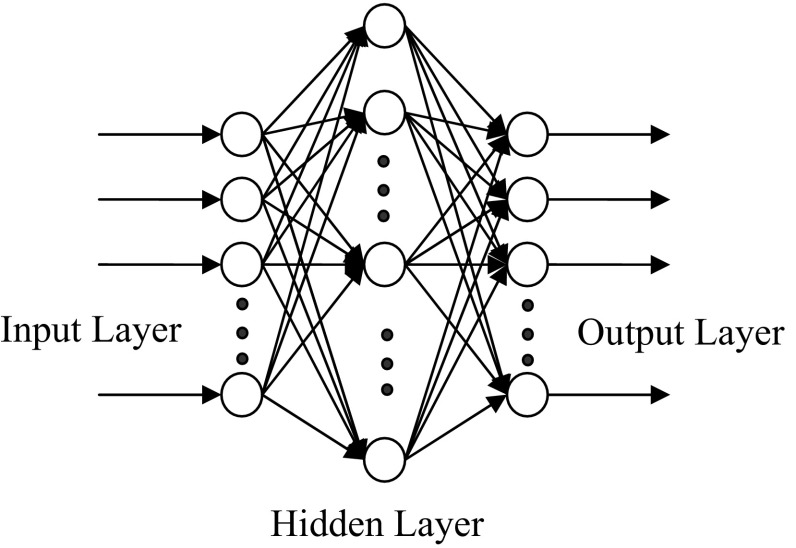
Perceptron structure with a hidden layer

The Back-Propagation Algorithm is one of Least Mean Square methods, which is normally used in engineering. In a multilayer perceptron, each neuron of a layer is linked to all neurons of the previous layer. Figure 2 shows a perceptron with a hidden layer. Each layer output acts as the input to the next neurons. To train Multilayer Feed Forward Neural Network, Back-Propagation Law is used. In the first stage, all weights and biases are selected according to small random numbers. In the second stage, input vector *X*_*p*_ = *x*_0_, *x*_1_, …, *x*_*n*−1_ and the target exit *T*_*p*_ = *t*_0_, *t*_1_, …, t_*m*−1_ are given to the network, where the subscripts n and m are the numbers of input and output vector, respectively. In the third stage, the following quantitative values are calculated and transferred to the subsequent layer until it eventually reaches the exit layer.

4 $$ O_{Pj} = f\left[ {\sum\limits_{i = 0}^{n - 1} {w_{i} x_{i} } } \right] $$

The fourth stage begins from the exit layer, during which the weight coefficients are corrected.

5 $$ w_{ij} (t + 1) = w_{ij} (t) + \eta \delta_{Pj} O_{Pj} $$

where “*W*_*ij*_(*t*)” stands for the weight coefficients from node “*i*” to node “*j*” in time “*t*”, “$\eta$” is the rate coefficient, “$\delta_{Pj}$” refers to the corresponding error of input pattern “*P*” to the node “*j*” and “$O_{Pj}$” is the output corresponding to the *j* neuron. “$\delta_{Pj}$” is calculated by the following equations for exit layer and hidden layer, respectively (Browne 1997):

6 $$ \delta_{Pj} = O_{Pj} (1 - O_{Pj} )(t_{Pj} - O_{Pj} ) $$

7 $$ \delta_{Pj} = O_{Pj} (1 - O_{Pj} )\sum\limits_{k} {\delta_{Pk} w_{jk} } $$

Here, the Σ acts for *k* nodes on the subsequent layer after the node “*j*”. In the learning process, there are several parameters that have influence on the ANN training. These parameters are the number of iterations, number of hidden layers and the number of hidden neurons. To find the best architecture of the model, best set of the aforementioned parameters based on minimizing the network output error should be chosen (Atashrouz et al. 2013, 2014a, b).

### Prediction of oxygen profile in a system containing wound dressing and skin

**Fig. 3:**
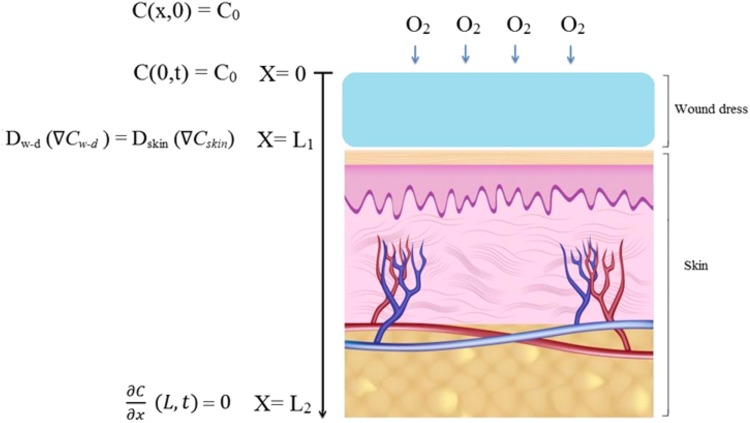
Schematic of wound dressing and skin with boundary conditions of system

Figure 3 shows a schematic of considered problem for prediction of oxygen concentration. As can be observed, the system is consisted of two regions of wound dressing and skin. In the wound dressing region, oxygen is only diffused and equation of continuity takes following form:

8 $$ \frac{\partial c}{\partial t} = (\nabla D_{w - d} \nabla c) $$

However, since in the skin layer due to existence of oxygen consumption by cells a reaction term should be added to equation:

9 $$ \frac{\partial c}{\partial t} = (\nabla D_{\text{skin}} \nabla c) - R $$

The reaction rate kinetic function describing the overall oxygen consumption for metabolic is assumed to has the Michaelis–Menten kinetics. In this regard equation takes following form:

10 $$ \frac{\partial c}{\partial t} = (\nabla D_{\text{skin}} \nabla c) - \frac{{v_{m} c}}{{k_{m} + c}} $$

where $$ v_{m} $$ and $$ k_{m} $$ are Michaelis–Menten parameters which $$ v_{m} $$ is the maximum rate of oxygen consumption and $$ k_{m} $$ is the concentration of oxygen when the rate of reaction is equal to $$ {\raise0.5ex\hbox{$\scriptstyle 1$} \kern-0.1em/\kern-0.15em \lower0.25ex\hbox{$\scriptstyle 2$}}v_{m} $$. The value of diffusion coefficient, specific oxygen consumption rate is equal to 2.54 × 10^−5^ cm^2^ s^−1^ and 3.33 × 10^−5^ s^−1^ respectively (Lee et al. 2013; von Heimburg et al. 2005).

The oxygen concentration at the upper boundary of the wound dressing is equal to atmospheric oxygen concentration (*C*_atm_) which has the following relation:

11 $$ C = C_{\text{atm}} ,\quad X = 0 $$

The equal flux condition exists at the interface of the wound dressing and skin which is as below:

12 $$ - D_{w - d} \nabla c\left| {_{w - d} } \right. = - D_{\text{skin}} \nabla c\left| {_{\text{skin}} } \right.,\quad X = L_{1} $$

where *L*_1_ is the thickness of wound dressing.

In addition, diffusion of oxygen to the right and left side of boundaries is equal to zero ($\nabla C = 0$).

## Results and discussion

### Modeling of the fiber diameter

To develop of GMDH and ANN models, concentration, flow rate, voltage, distance, and speed of collector were used as input parameter and diameter of fibers was considered as output parameter. Variable parameters of electrospinning are tabulated in Table 1. Optimized GMDH model was obtained based on the experimental data and tabulated in Table 2. In addition, it should be noted that experimental data were divided into two sections: randomly, 70 % of experimental data were used for training the model and the rest 30 % were considered for testing the model. It should be emphasized that test data set is necessary because in the proposed model, maybe performance for estimation of train data would be satisfactory, but when the model is applied for a new sample the result is not accomplished with good accuracy.

**Table 1:** Characteristic of different samples

Number of sample	Concentration (%)	Flow rate (cc/h)	Voltage (KV)	Distance (cm)	Speed of collector (rpm)	Diameter (nm)
1	10	0.1	20	12	200	134.19
2	10	0.3	20	12	200	168.49
3	10	0.4	20	12	200	185.35
4	10	0.6	20	12	200	226.74
5	10	0.4	20	12	800	167.71
6	10	0.4	20	12	1500	159.96
7	10	0.4	20	12	2500	159.09
8	10	0.3	22	12	800	188.46
9	10	0.3	20	12	1500	158.09
10	10	0.3	20	12	2500	156.35
11	10	0.4	15	10	200	158.62
12	10	0.4	17.5	10	200	175.19
13	10	0.4	20	10	200	182.91
14	10	0.4	22.5	10	200	203.69
15	10	0.4	20	8	200	183.65
16	10	0.4	22	8	200	192.79
17	10	0.4	24	8	200	215.31
18	10	0.4	26	8	200	216.76
19	12	0.3	19	12	600	217.42
20	12	0.4	22	12	600	250.26
21	12	0.5	22	12	600	259.33
22	12	0.6	22	12	600	260.93
23	12	0.5	23	12	600	259.90
24	12	0.5	25	12	600	268.60
25	12	0.6	25	12	600	277.80
26	12	0.2	12	12	400	197.91
27	12	0.2	16	12	400	203.50
28	12	0.2	26	12	400	219.75
29	12	0.2	30	12	400	263.26

**Table 2:** Mathematical relation for GMDH model

Layer 1
$$ N_{1} = 57.899 - 0.978409X_{4} - 0.0118487X_{5} + 502124X_{1}^{4} + 144.964X_{2} + 0.0842974X_{3}^{2} $$
Output layer
$$ d = - 84.2446 + 0.00342148X_{5} - 24.1294X_{2} + 100.383X_{2}^{4} + 1.65187N_{1} - 4.80264 \times 10^{ - 6} N_{1}^{3} $$

*X*_1_, concentration; *X*_2_, flow rate; *X*_3_, voltage; *X*_4_, distance; *X*_5_ speed of collector

**Fig. 4:**
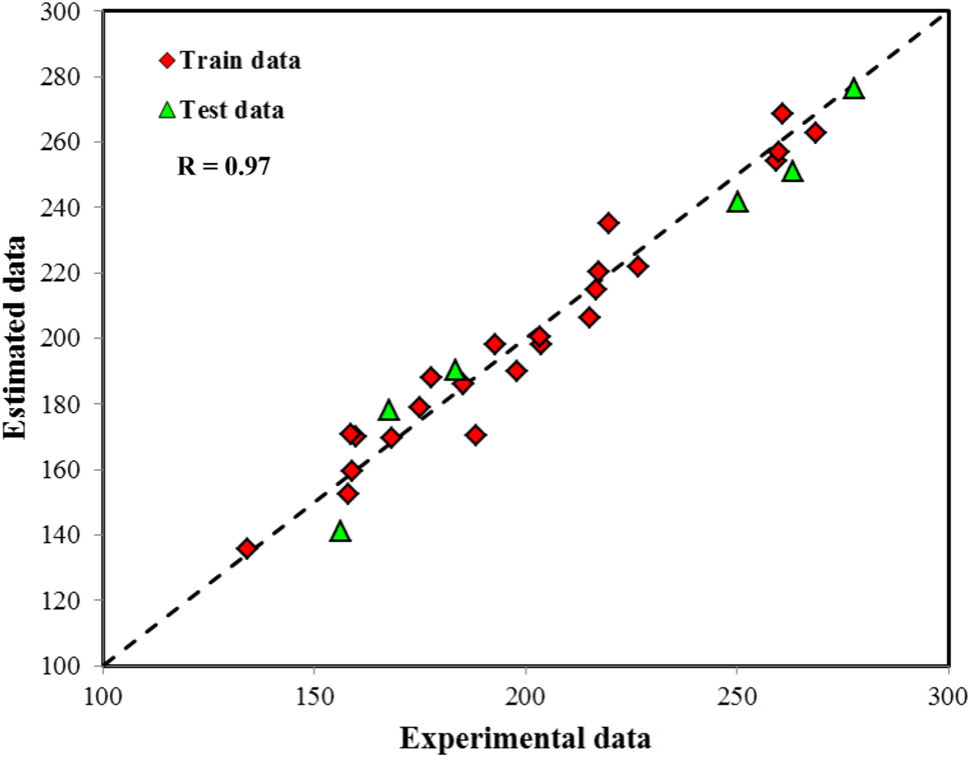
Comparison between estimated data by GMDH model and experimental data

**Fig. 5:**
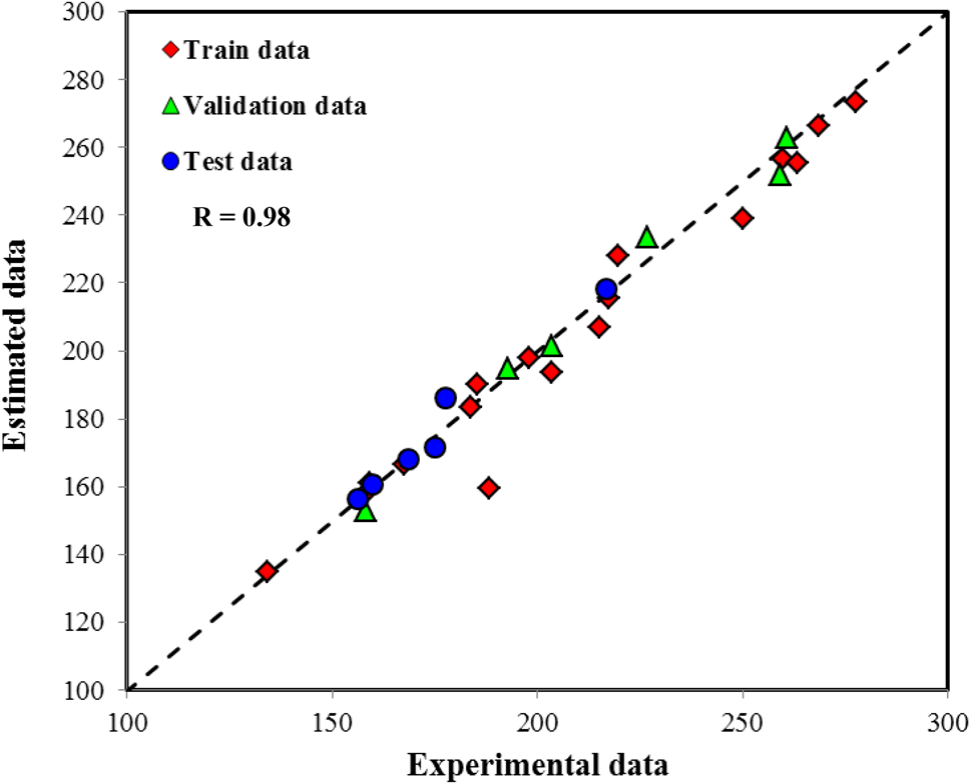
Comparison between estimated data by ANN model and experimental data

Figures 4 and 5 shows estimated data by GMDH and ANN models as experimental data. Both models indicate good performance and experimental data are keep close to diagonal line. The average absolute relative deviation for GMDH and ANN models was equal to 3.56 and 2.28 %, respectively. The average absolute relative deviation for model is defined as follows:

13 $$ {\text{MARD}}\% = \frac{1}{100}\sum\limits_{i = 1}^{N} {\frac{{\left| {d_{\text{experimental}} - d_{\text{estimation}} } \right|}}{{d_{\text{experimental}} }}} $$

**Fig. 6:**
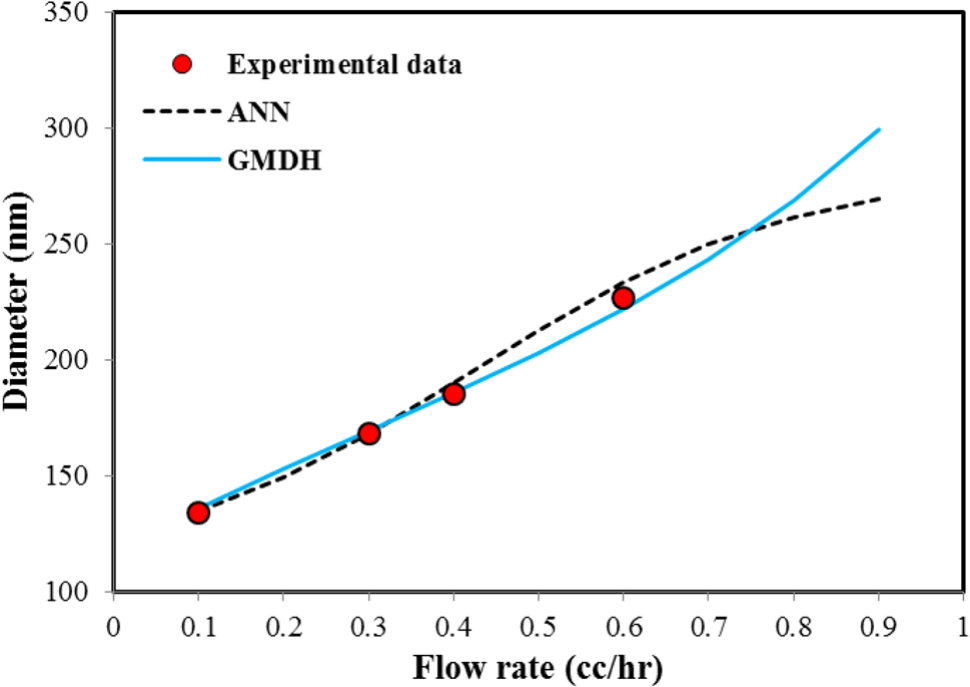
The effect of flow rate variations on nanofiber diameter and prediction of GMDH and ANN models (concentration 10 %, voltage 20 kV, collection distance 12 cm and collector speed 200 rpm)

Comparing the MARD% of both models indicates that ANN is more accurate than GMDH. In addition to the good accuracy for both models, it will be interesting to check the physical response of the models to different parameters. It means, as explained in previous section when all input parameters expect one of them are constant, the diameter of fibers can increase or decrease with changing the considered parameter. To check this possibility for GMDH and ANN models, the effect of various parameters on response of both models was considered. Figure 6 represents the effect of flow rate on the nanofiber diameter and the predicted results by GMDH and ANN models. As shown in this figure, both GMDH and ANN models have well prediction about increased diameter and desired behavior. However, the results of ANN model for higher flow rate of 0.8 cc/h are different from GMDH model. It should be noted that according to existing theories of neural networks, this type of model cannot be extrapolated and just used for the modeling and interpolation between the experimental data. This means that it is better to use the neural networks in the range of variables to reduce the possibility of incorrect predictions.

**Fig. 7:**
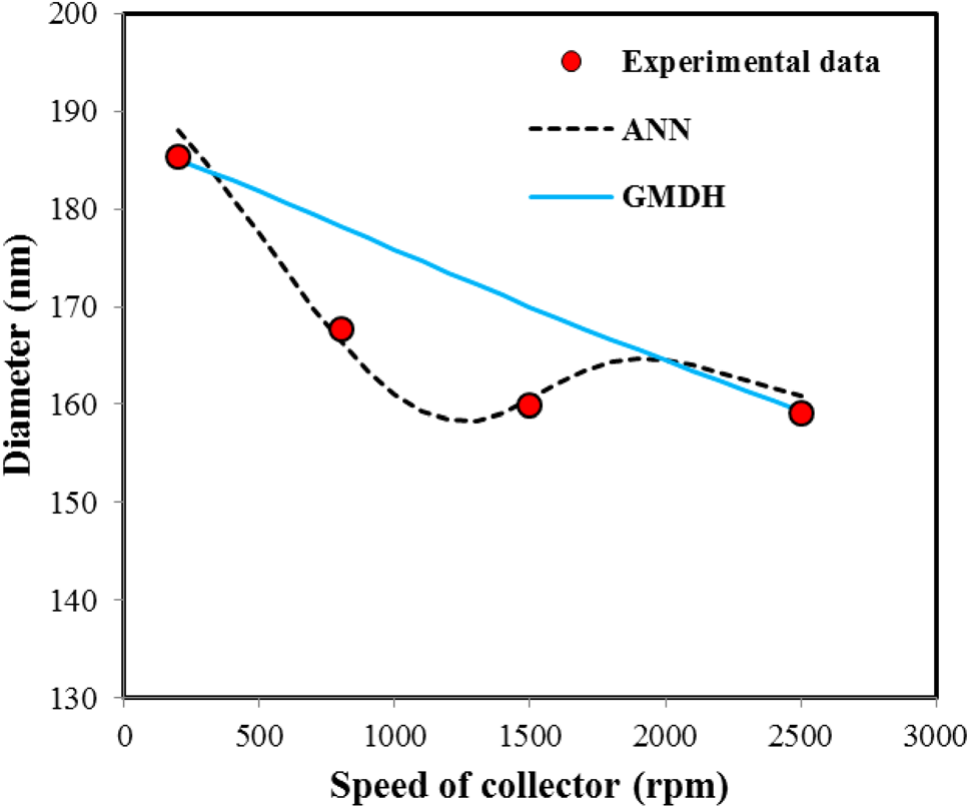
The effect of speed of collector variations on nanofiber diameter and prediction of GMDH and ANN models (concentration 10 %, flow rate 0.4 cc/h, voltage 20 kV and collection distance 12 cm)

Figure 7 shows variations of diameter as speed of collector. As can be seen GMDH model was predicted a descending trend with increasing the speed of collector, nanofiber diameter decreased, but ANN model shows different slop trend. Thus, it can be concluded, ANN model has a good ability to conformity on the experimental data, but physically this model is not able to predict the trend of decreasing the nanofiber diameter.

**Fig. 8:**
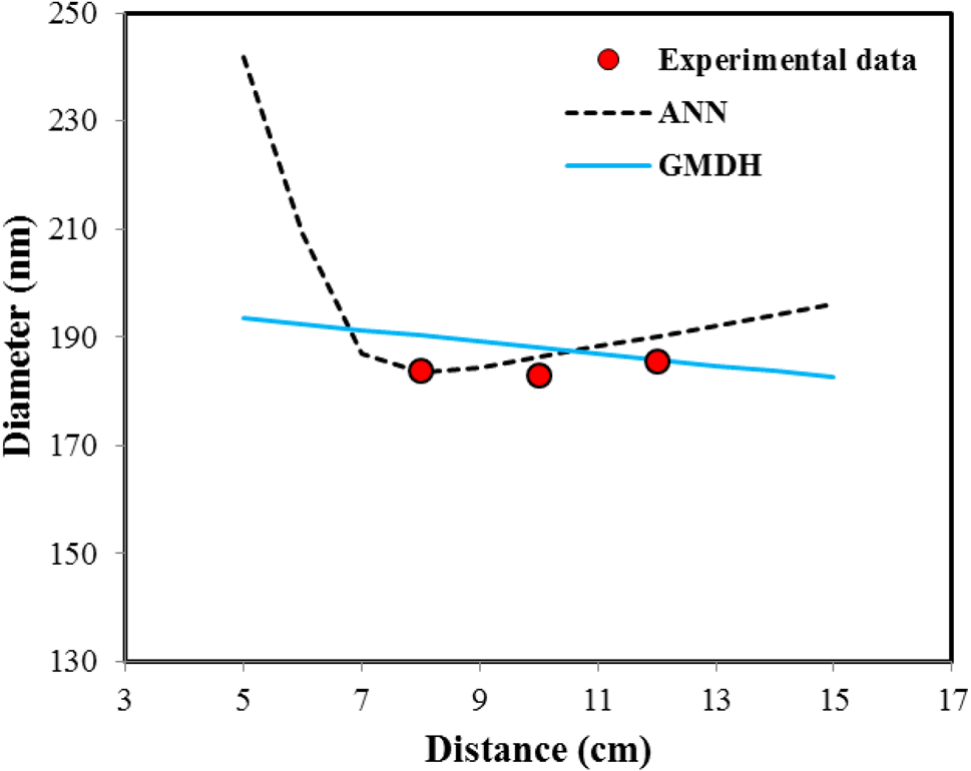
The effect of distance variations on nanofiber diameter and prediction of GMDH and ANN models (concentration 10 %, flow rate 0.4 cc/h, voltage 20 kV and collector speed 200 rpm)

In addition Fig. 8 shows variations of diameter as collector distance. According to this figure, ANN model shows irrational prediction and with increasing distance, nanofiber diameter first decrease and then increase, but GMDH model as well predict the behavior of experimental data, but have some deviations with them. This is due to the type of functions is intended to develop the GMDH model. In addition, another advantage of GMDH model in comparison to ANN model is its simple mathematical structure. While, ANNs have complex mathematical form which make their applicability difficult.

### Experimental investigation of the electrospinning parameters on fiber diameter

**Fig. 9:**
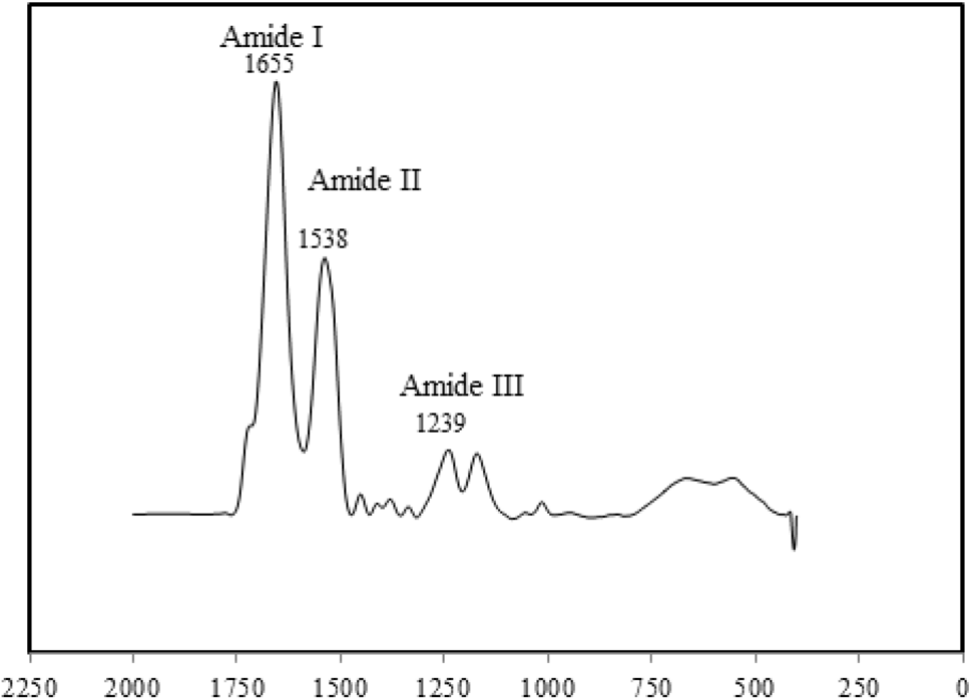
FTIR spectra of silk fibroin mats

Figure 9 shows FTIR spectroscopy of regenerated silk fibroin and as expected from previous works peaks in the Amide I (1655 cm^−1^), Amide II (1538 cm^−1^) and Amide III (1239 cm^−1^) related to random coil structure of it are observed (Rousseau et al. 2004; Mirahmadi et al. 2013).

**Fig. 10:**
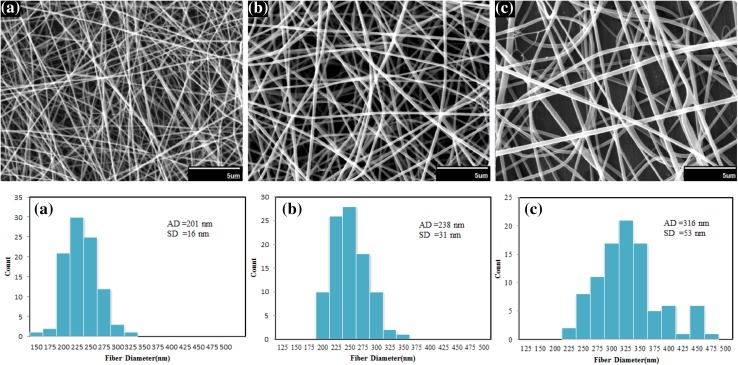
SEM images and diameter distributions of electrospun nanofibers of silk fibroin prepared from silk fibroin aqueous solution under different concentrations: **a** 10 %, **b** 12 %, **c** 13 % (flow rate 0.3 cc/h, voltage 24 kV and collection distance 12 cm). *Scale bars* 5 µm

The SEM images of electrospun silk nanofibers under different concentrations are given in Fig. 10. It is seen that increasing in polymer concentration from 10 to 13 % results in fiber diameter increase ranging from 135 ± 16 nm to about 300 ± 29 nm.

This can be explained by two reasons: first, increase in the amount of polymer in electrospinning jet and second, more interaction between polymer chains in solution which later is lead to more resistance of solution against pulling by electrical charges. This behavior is also observed in the study of Sukigara et al. (2004).

**Fig. 11:**
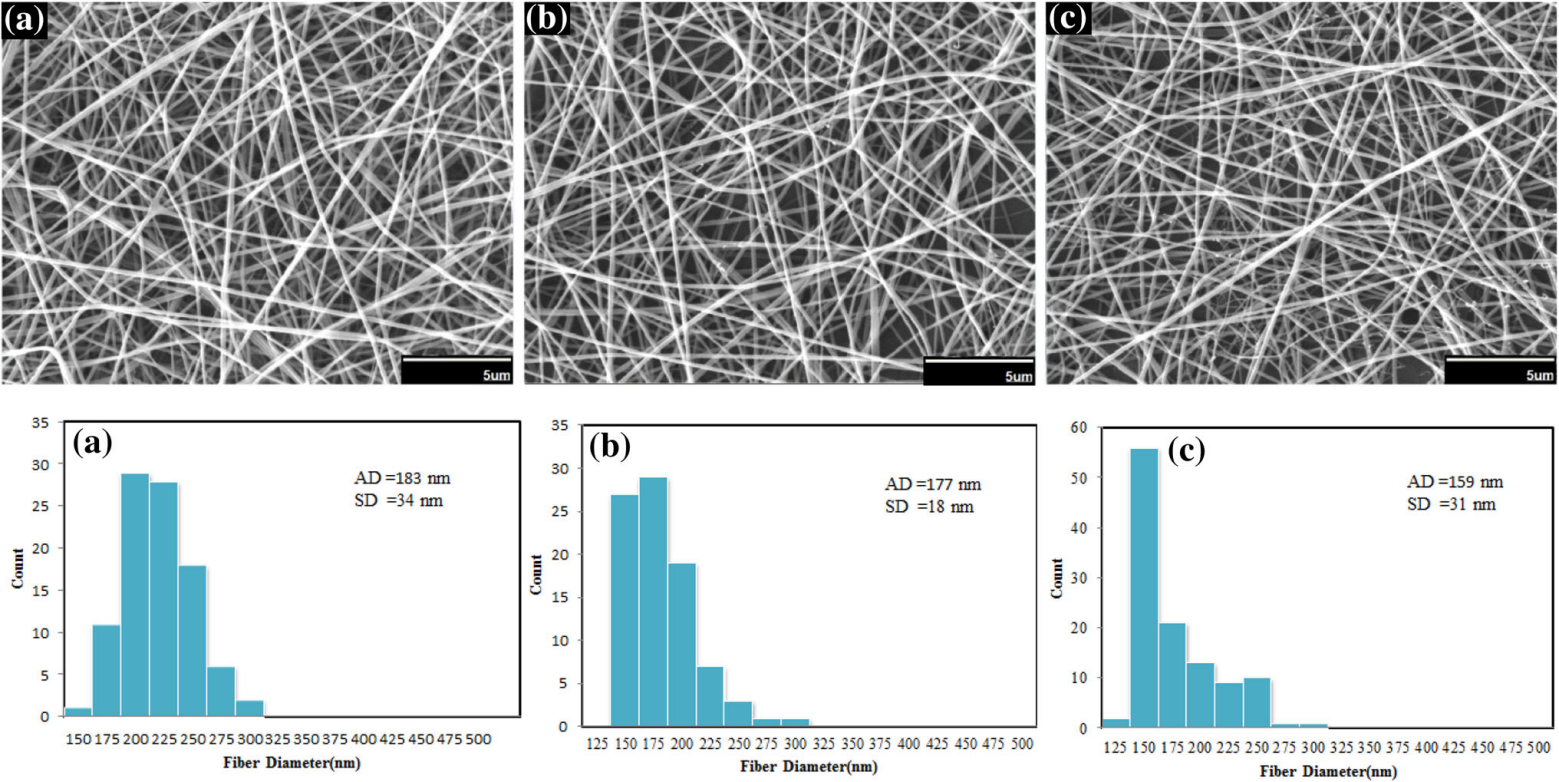
SEM images and diameter distributions of electrospun nanofibers of silk fibroin under different collection distances: **a** 8 cm, **b** 10 cm, **c** 12 cm (concentration 10 %, voltage 20 kV, and flow rate 0.4 cc/h). *Scale bars* 5 µm

Figure 11 shows SEM images of electrospun nanofibers of silk fibroin under different spinning distances. If other parameters were kept constant, increasing of spinning distance is lead to more evaporation of solvent and consequently decreases in diameter of fibers slightly. It should be noted that the morphology of electrospun nanofibers is not changed strongly. So spinning distance is not considered very effective parameter in the morphology of electrospun nanofibers (Sukigara et al. 2004; Zhou et al. 2009).

**Fig. 12:**
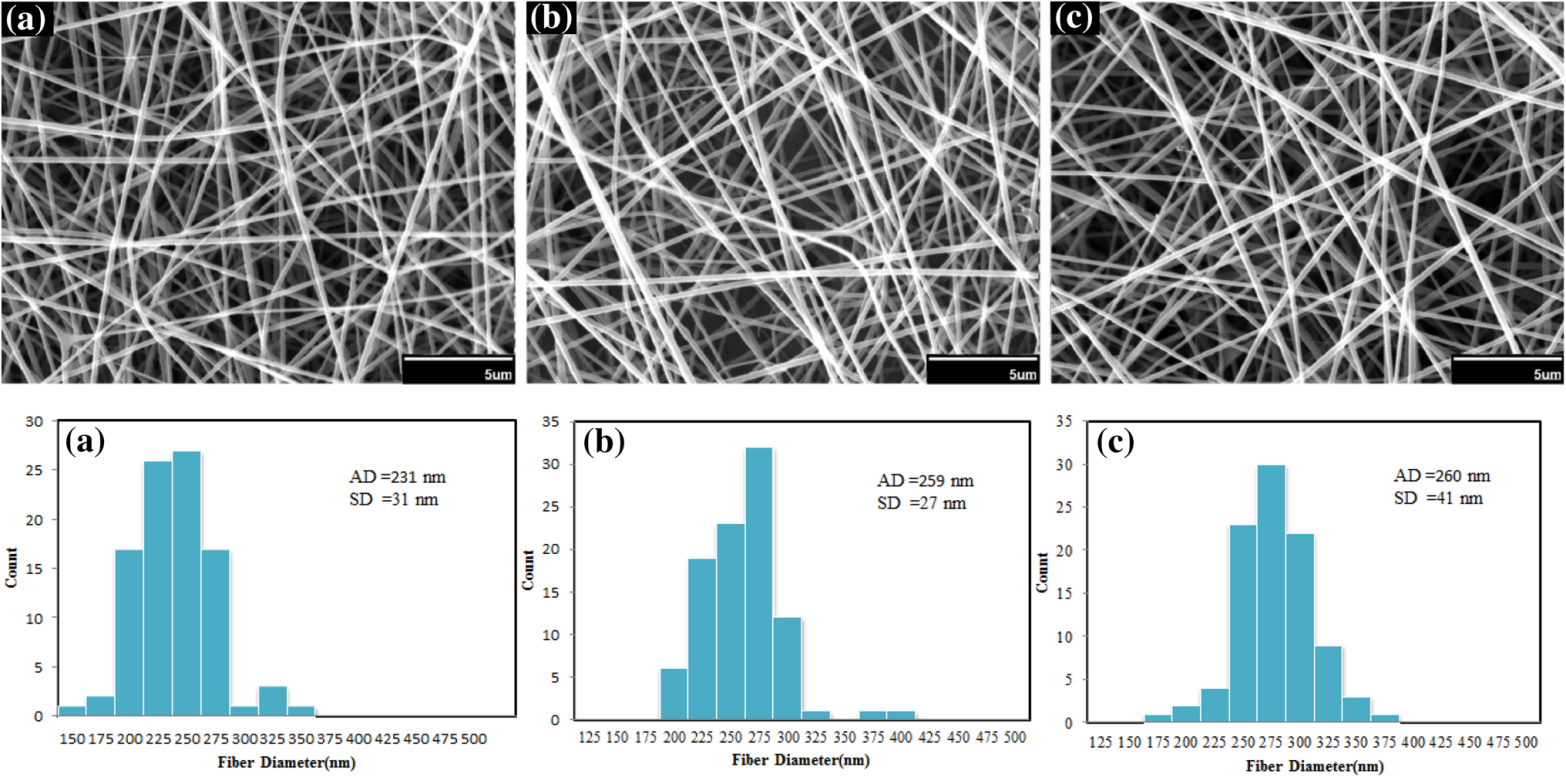
SEM images and diameter distributions of electrospun nanofibers of silk fibroin under different flow rates: **a** 0.3 cc/h, **b** 0.5 cc/h, **c** 0.6 cc/h (concentration 12 %, voltage 22 kV and collection distance 12 cm). *Scale bars* 5 µm

Figure 12 shows the effect of increase in flow rate on fiber diameter. Obviously with increase in the flow rate, more volume of solution is exit from the needle. Therefore, diameter of the fibers is increased. The flow rate has great importance effect on the formation of Taylor’s cone and with increase of flow rate is caused to uniform morphology of silk fibroin nanofibers. Consequently, flow rate is a key parameter for obtaining an appropriate structure in electrospinning (Megelski et al. 2002).

### Oxygen profile in wound dressing and skin

Available analytical methods are not capable to solve the continuity equation when reaction rate of Michaelis–Menten kinetic is presented. Consequently, to solve equations and boundary conditions we should use numerical methods. In this regard, implicit finite difference method was considered for solving the problem and programming was conducted in MATLAB software. It should be noted that parameter of Michaelis–Menten kinetic was used from refs (Lee et al. 2013; von Heimburg et al. 2005).

**Fig. 13:**
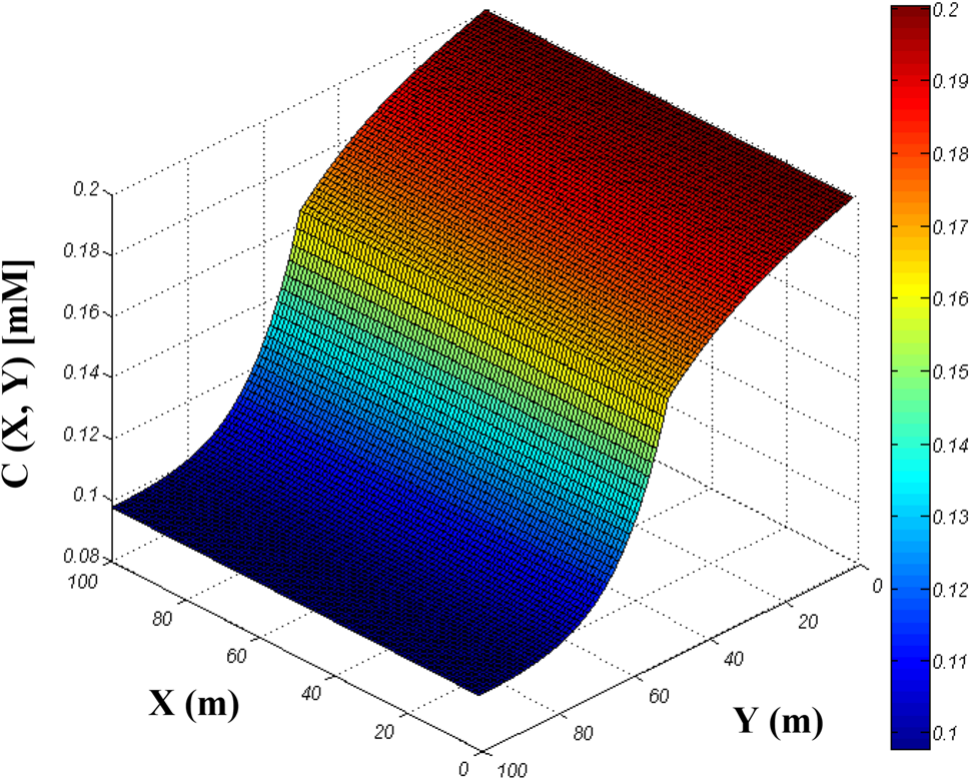
Three-dimensional oxygen profile in wound dressing and skin system (relationship between oxygen concentration and skin + wound dressing)

The result of solving the equations is presented in Fig. 13. The figure is shown the oxygen profile after 1 day that wound dress is attached to the skin. As can be seen, oxygen can be suitably diffuse on the wound dress and reach to the skin layer for consumption by cells. It should be noted that last layer of skin is more sensitive to the oxygen because the magnitude of oxygen has its lower value in this region. The lower magnitude of oxygen in last layer of skin is logical because the problem is diffusion control. It means the only mechanism to transport oxygen is diffusion mass transfer which should be contest with the oxygen consumption of cells. However, the figure shows that diffusion of oxygen in our proposed wound dress is appropriate for transporting of oxygen to even the last layer of skin.

## Conclusion

In the present study, electrospinning of silk fibroin-based nanofibrous mat under different conditions was performed and effect of various parameters on the diameter of nanofibers was investigated. For example, we observed that increasing the solution concentration from 10 to 13 % results in increasing fiber diameters from 135 ± 16 nm to about 300 ± 29 nm. Oppositely, increasing distance from 8 cm to 12 cm results in fiber diameters decreasing from 180 ± 31 to 158 ± 23 nm. Furthermore, two mathematical models based on the GMDH and ANN were developed for prediction of nanofibers diameter. Both models have good accuracy (MARD% = 3.56 % for GMDH and MARD% = 2.28 % for ANN model), ANN model has less error than GMDH model, but it has not good ability to predict the trend of increase and decrease of diameter with variation of electrospinning parameters. So GMDH model is more appropriate for modeling the similar research study. In addition, a mathematical model was proposed for prediction of oxygen profile in wound dressing which it can be concluded that the problem is diffusion control. In addition, the rate of oxygen consumption by cells is more than transported equation with diffusion mechanism.

## References

[CR1] Atashrouz S, Mirshekar H, Bagheri H (2013). Correlation of vapor–liquid equilibria for commonly used binary systems in supercritical fluid extraction processes. Int J Sci Eng.

[CR2] Atashrouz S, Amini E, Pazuki G (2014). Modeling of surface tension for ionic liquids using group method of data handling. Ionics.

[CR3] Atashrouz S, Pazuki G, Alimoradi Y (2014). Estimation of the viscosity of nine nanofluids using a hybrid GMDH-type neural network system. Fluid Phase Equilib.

[CR4] Atashrouz S, Mozaffarian M, Pazuki G (2015). Modeling the thermal conductivity of ionic liquids and ionanofluids based on group method of data handling and modified Maxwell model. Ind Eng Chem Res.

[CR5] Atashrouz S, Pazuki G, Kakhki SS (2015). A GMDH-type neural network for prediction of water activity in glycol and Poly (ethylene glycol) solutions. J Mol Liq.

[CR6] Browne A (1997). Neural network analysis, architectures and applications.

[CR7] Chirila TV, Suzuki S, Bray LJ, Barnett NL, Harkin DG (2013). Evaluation of silk sericin as a biomaterial: in vitro growth of human corneal limbal epithelial cells on *Bombyx mori* sericin membranes. Prog Biomater.

[CR8] Dodangeh M, Gharanjig K, Arami M, Atashrouz S (2014). Surface alteration of polyamide fibers by polyamidoamine dendrimers and optimization of treatment process using neural network towards improving their dyeing properties. Dyes Pigm.

[CR9] Hromadka M, Collins JB, Reed C, Han L, Kolappa KK, Cairns BA, Andrady T, van Aalst JA (2008). Nanofiber applications for burn care. J Burn Care Res.

[CR10] Jin H-J, Park J, Karageorgiou V, Kim U-J, Valluzzi R, Cebe P, Kaplan DL (2005). Water-stable silk films with reduced β-sheet content. Adv Funct Mater.

[CR11] Kim SH, Nam YS, Lee TS, Park WH (2003). Silk fibroin nanofiber. Electrospinning, properties, and structure. Polym J.

[CR12] Kim HJ, Kim HS, Matsumoto A, Chin I-J, Jin H-J, Kaplan DL (2005). Processing windows for forming silk fibroin biomaterials into a 3D porous matrix. Aust J Chem.

[CR13] Lee SY, Lee BR, Lee J, Kim S, Kim JK, Jeong YH, Jin S (2013). Microscale diffusion measurements and simulation of a scaffold with a permeable strut. Int J Mol Sci.

[CR14] Malallah A, Nashawi IS (2005). Estimating the fracture gradient coefficient using neural networks for a field in the Middle East. J Pet Sci Eng.

[CR15] Megelski S, Stephens JS, Chase DB, Rabolt JF (2002). Micro-and nanostructured surface morphology on electrospun polymer fibers. Macromolecules.

[CR16] Min B-M, Lee G, Kim SH, Nam YS, Lee TS, Park WH (2004). Electrospinning of silk fibroin nanofibers and its effect on the adhesion and spreading of normal human keratinocytes and fibroblasts in vitro. Biomaterials.

[CR17] Mirahmadi F, Tafazzoli-Shadpour M, Shokrgozar MA, Bonakdar S (2013). Enhanced mechanical properties of thermosensitive chitosan hydrogel by silk fibers for cartilage tissue engineering. Mater Sci Eng, C.

[CR18] Nasouri K, Bahrambeygi H, Rabbi A, Shoushtari AM, Kaflou A (2012). Modeling and optimization of electrospun PAN nanofiber diameter using response surface methodology and artificial neural networks. J Appl Polym Sci.

[CR19] Rousseau M-E, Lefevre T, Beaulieu L, Asakura T, Pézolet M (2004). Study of protein conformation and orientation in silkworm and spider silk fibers using Raman microspectroscopy. Biomacromolecules.

[CR20] Sukigara S, Gandhi M, Ayutsede J, Micklus M, Ko F (2004). Regeneration of *Bombyx mori* silk by electrospinning. Part 2. Process optimization and empirical modeling using response surface methodology. Polymer.

[CR21] Uttayarat P, Jetawattana S, Suwanmala P, Eamsiri J, Tangthong T, Pongpat S (2012). Antimicrobial electrospun silk fibroin mats with silver nanoparticles for wound dressing application. Fibers Polym.

[CR22] Valenzuela F, Covarrubias C, Martínez C, Smith P, Díaz-Dosque M, Yazdani-Pedram M, Bellucci D, Sola A, Gentile P, Ciardelli G (2012). Bionanocomposites based on hydroxyapatite and bioactive glass nanoparticles. J Biomed Mater Res B Appl Biomater.

[CR23] Von Heimburg D, Hemmrich K, Zachariah S, Staiger H, Pallua N (2005). Oxygen consumption in undifferentiated versus differentiated adipogenic mesenchymal precursor cells. Respir Physiol Neurobiol.

[CR24] Wang M, Jin H-J, Kaplan DL, Rutledge GC (2004). Mechanical properties of electrospun silk fibers. Macromolecules.

[CR25] Wharram SE, Zhang X, Kaplan DL, McCarthy SP (2010). Electrospun silk material systems for wound healing. Macromol Biosci.

[CR26] Zhang G, Patuwo BE, Hu MY (1998). Forecasting with artificial neural networks: the state of the art. Int J Forecast.

[CR27] Zhou J, Cao C, Ma X (2009). A novel three-dimensional tubular scaffold prepared from silk fibroin by electrospinning. Int J Biol Macromol.

